# New species and new records of the genus *Deinopteroloma* Jansson, 1946 (Coleoptera, Staphylinidae, Omaliinae) from China

**DOI:** 10.3897/zookeys.846.32568

**Published:** 2019-05-16

**Authors:** Zhi-Fei Cheng, Liang Tang, Li-Zhen Li, Zhong Peng

**Affiliations:** 1 Department of Biology, College of Life Sciences, Shanghai Normal University, Shanghai, 200234, P. R. China Shanghai Normal University Shanghai China

**Keywords:** Coleoptera, Staphylinidae, Omaliinae, *
Deinopteroloma
*, China, taxonomy, new species, new records

## Abstract

New morphological, taxonomic and faunistic data of the genus *Deinopteroloma* Jansson, 1946 from China are provided. Two species are described and illustrated: *D.songi* Peng & L.-Z. Li, **sp. n.** (Xizang: Pailong) and *D.spinigerum* Peng & L.-Z. Li, **sp. n.** (Hunan: Mangshan). New provincial records are provided for *D.hamatum* Smetana, 1996 from Anhui, Zhejiang and Jiangxi, *D.obtortum* Assing, 2015 from Sichuan, and *D.tricuspidatum* Smetana, 1996 from Zhejiang.

## Introduction

New morphological, taxonomic and faunistic data on the genus *Deinopteroloma* Jansson, 1946 includes 26 species distributed in Palaearctic and Oriental regions, with 12 of them known from China. The *Deinopteroloma* fauna of Sichuan with eight described species is currently the most diverse of all Chinese provinces (Assing 2015; Shavrin and Smetana 2016).

In recent years, we obtained numerous *Deinopteroloma* specimens collected during several field trips. Five species were identified, two of which are described and illustrated in the present study.

## Material and methods

The examined material is deposited in the following public collections:

**SNUC** Insect Collection of Shanghai Normal University, Shanghai, China

**CNC** Canadian National Collection of Insects, Ottawa, Canada

The genitalia and other dissected parts were mounted on plastic slides and attached to the same pin as the respective specimens. Photographs were taken with a Canon EOS 7D camera with a MP-E 65 mm macro lens or with a Canon G9 camera mounted on an Olympus CX31 microscope.

The following abbreviations were used in the text, with all measurements in millimeters:

**BL** Body length: length of body from apices of mandibles to abdominal apex.

**FL** Forebody length: length of forebody from the anterior margin of the mandibles to the posterior margin of the elytra.

**HL** Head length: length of head from anterior margin of frons to posterior constriction of head.

**HW** Head width: maximum width of head.

**AnL** Antenna length: length of antenna from base of antennomere I to apex of antennomere XI.

**PL** Pronotum length: length of pronotum along midline.

**PW** Pronotum width: maximum width of pronotum.

**EL** Elytral length: length at suture from apex of scutellum to posterior margin of elytra.

**EW** Elytral width: combined width of elytra.

**AL** Length of aedeagus: length of aedeagus from apex of longer paramere to base of aedeagal capsule.

The type labels were cited with the original spelling; different labels are separated by slashes.

## Results

### Deinopteroloma hamatum

Taxon classificationAnimaliaColeopteraStaphylinidae

Smetana, 1996

(Figs 1 , 2A–D , 5)

Deinopteroloma hamatum Smetana, 1996: 79.
Deinopteroloma hamatum : Smetana 2001: 57, Shavrin and Smetana, 2016: 227.

#### Type material examined.

Paratype ♂ [teneral]: “KUATUN FUKIEN, China 21.4.46, (TSCHUNG SEN.) / PARATYPUS, *Deinopterolomahamatum*, A. Smetana 1995 [yellow label]” (CNC).

#### Additional material examined.

(9 ♂♂, 6 ♀♀). **China: Anhui**: 2 ♂♂, 2 ♀♀, Huang Shan, Jiulongpu, 30°06'N, 118°12'E, 460–910 m, 26.XI.2011, Zhong Peng leg. (SNUC); 1 ♂, 1 ♀, Guniujiang, 360–420 m, 30.IV.2005, Tang & Hu leg. (SNUC); 2 ♂♂, Guniujiang, 950–1050 m, 28.IV.2005, Tang & Hu leg. (SNUC). **Zhejiang**: 1 ♂, Anji, Longwang Shan, 950–1200 m, 25.VI.2004, Tang & Hu leg. (SNUC); 1 ♀, Anji, Longwang Shan, 350–550 m, 24.VI.2006, Jin-Wen Li leg. (SNUC); 1 ♂, Anji, Longwang Shan, Qianmutian, 1450 m, 30°23'N, 119°26'E, 14.V.2013, Li & Zheng leg. (SNUC); 1 ♂, 1 ♀, Zhuji, Dongbai Shan, 300 m, 31.III.2013, Tie-Xiong Zhao leg. (SNUC); 1 ♂, Qingyuan County, Baishanzu, 1700 m, 27°45'25"N, 119°12'06"E, 2.V.2014, Zhong Peng leg. (SNUC); **Jiangxi**: 1 ♀, Sanqing Shan, 800 m, 4.V.2005, Tang & Hu leg. (SNUC).

#### Comparative notes.

**Figure 1.**
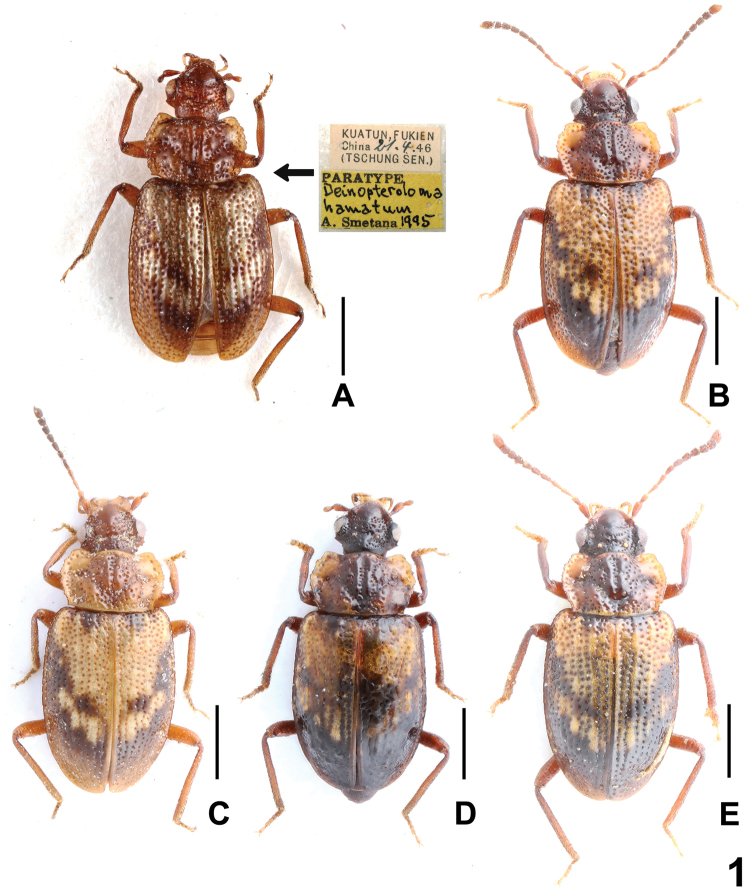
Habitus of *Deinopterolomahamatum*: **A** paratype, with type labels, **B–E** male (**B** Anhui; **C–E** Zhejiang). Scale bars: 1.0 mm.

**Figure 2.**
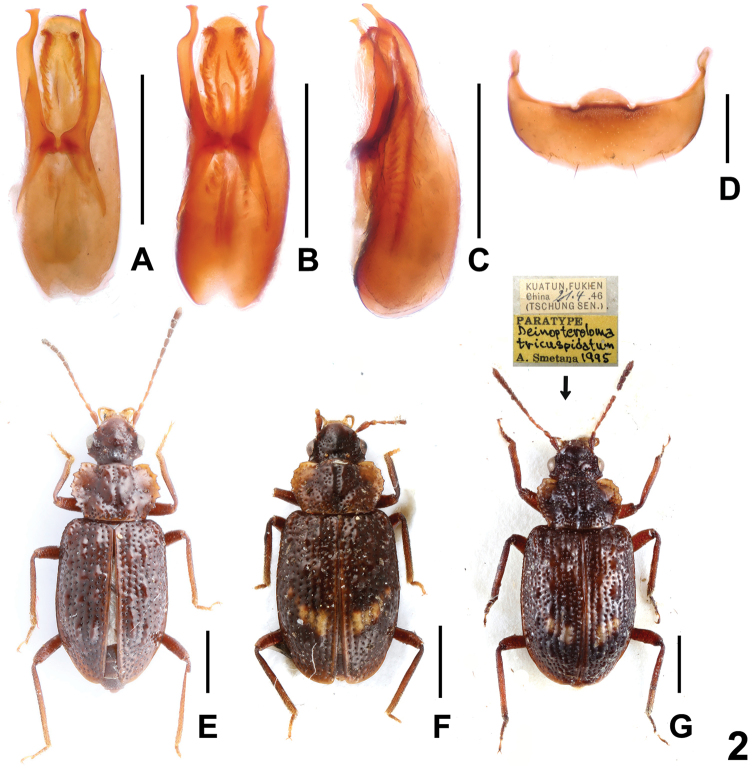
*Deinopterolomahamatum* (**A–D**): **A–B** aedeagus in ventral view, **C** aedeagus in lateral view, **D** male sternite VIII. Habitus of *Deinopteroloma* spp (**E–G**): **E***D.obtortum*, **F***D.tricuspidatum*, **G** paratype of *D.tricuspidatum*, with type labels. Scale bars: 0.5 mm(**A–D**), 1.0 mm (**E–G**).

The original description was based on three type specimens from Guadun, Fujian (Smetana 1996). Considerable interspecific variability of *D.hamatum* was observed not only in external characters such as body size, coloration, punctation, and shapes of head, pronotum and elytra (Fig. 1), but also in the shape of parameres and internal structures of aedeagus (Fig. 2A, B). The shape of the posterior margin of the male tergite VIII is constant (Fig. 2D). It is recorded here from Anhui, Zhejiang and Jiangxi for the first time. Some specimens were sifted from wet leaves and moss on the rocks near a stream at altitudes from about 460 to 910 m (Fig. 5).

### Deinopteroloma obtortum

Taxon classificationAnimaliaColeopteraStaphylinidae

Assing, 2015

(Figs 2E , 6)

Deinopteroloma obtortum Assing, 2015: 1225.
Deinopteroloma obtortum : Shavrin and Smetana, 2016: 228.

#### Material studied.

**China: Sichuan**: 1 ♂, Xiaojin County, Jiajin Shan, 30°48'49"N, 102°42'55"E, 2490 m, 30.VII.2016, Zhou, Jiang, Liu & Gao leg. (SNUC).

#### Comment.

The original description was based on two type specimens from Min Shan, Gansu (Assing 2015). It is recorded from Sichuan for the first time. For illustrations of *D.obtortum* see figure 2E and figures 7–8, 15–18, 23–25 in Assing (2015). The specimen was sifted from leaf litter near the mountain track in mixed deciduous forests at an altitude of 2490 m (Fig. 6).

### Deinopteroloma songi

Taxon classificationAnimaliaColeopteraStaphylinidae

Peng & L.-Z. Li sp. n.

http://zoobank.org/E941914F-A912-4E89-9C78-C125CC234D4D

(Figs 3 , 7)

#### Type material.

Holotype ♂: China: Xizang Prov., Linzhi County, Pailong, 30°02'N, 95°00'E, 13.IV.2017 2100 m, Xiao-Bin Song leg. / Holotypus ♂ *Deinopterolomasongi* sp. n., det. Peng & Li. 2018 (SNUC). Paratypes: 6 ♂♂, 10 ♀♀: same label data as holotype / PARATYPE (yellow), *D.songi* sp. nov., det. Peng & Li, 2019 (SNUC).

#### Description.

Measurements (in mm) and ratios: BL 3.50–3.73, FL 3.44–3.65, HL 0.59–0.67, HW 0.78–0.83, AnL 1.68–1.78, PL 0.83–0.93, PW 1.30–1.47, EL 1.92–2.09, EW 1.50–1.67, AL 0.98–1.08, HL/HW 0.76–0.87, HW/PW 0.56–0.64, HL/PL 0.71–0.77, PL/PW 0.63–0.68, EL/EW 1.25–1.29.

**Figure 3.**
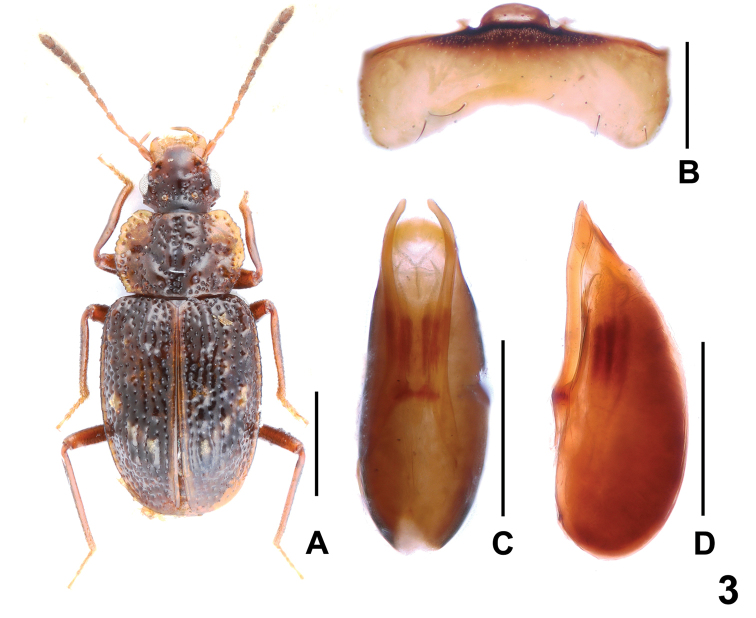
*Deinopterolomasongi* sp. n. **A** Habitus of paratype, **B** male sternite VIII, **C** aedeagus in ventral view, **D** aedeagus in lateral view. Scale bars: 1.0 mm (**A**), 0.5 mm (**B–D**).

Habitus as in Fig. 3A. Coloration: Body dark-brown; pronotum with broadly dark-yellowish lateral margins; each elytron with 3 or 4 yellowish brown tubercles; antenna with antennomeres I–IV yellowish and V–VIII gradually more infuscate apically, VIII–XI blackish-brown; maxillary palpi dark-yellowish.

Head transverse; posterior part of clypeus and vertex distinctly elevated, with indistinct lateral impressions in middle part and semicircular deep impression in front of ocelli, infraorbital ridges slightly impressed; eyes large and convex; small acute postocular ridge situated away from posterior margin of eye; ocelli large; frons smooth and glossy. All antennomeres longer than broad; measurements of antennomeres (length): I: 0.22; II: 0.14; III–VII: 0.16; VIII: 0.13; IX–X: 0.11; XI: 0.19.

Pronotum distinctly transverse; lateral margins finely crenulate and with somewhat regular outline; lateral portions strongly impressed; disc of pronotum with wide, very convex middle elevation and with distinct longitudinal impression; punctation coarse, rather dense in posteromedian portions, sparser in anteromedian and lateral portions, and very irregular and scattered in median portion.

Elytra without distinct longitudinal ridges; suture elevated in posterior two thirds; punctation coarse and arranged in partly irregular series (except in posterior portion of elytra); each elytron with approximately 3 or 4 smooth tubercles. Hind wings present.

Male. Protarsomeres I–IV very weakly dilated; sternites III–VII unmodified; sternite VIII (Fig. 3B) distinctly transverse, posterior margin broadly concave in middle; aedeagus as in Fig. 3C, D, both parameres slender and slightly curved in ventral view, distinctly extending apex of median lobe; internal sac with pair of sclerotized spines.

Female. Protarsomeres I–IV not dilated. Abdominal sternite VIII without posterior excision. Otherwise similar to male.

#### Comparative notes.

Based on the coloration of the body, 6–8 smooth tubercles of the elytra, the presence of pronounced elevations on the pronotum and elytra, as well as the morphology of the aedeagus, the new species is most similar to *D.sextuberculatum* Shavrin & Smetana, 2016, from which it can be distinguished by the darker coloration, the smaller size (*D.sextuberculatum*: 4.25–4.92 mm), the shape of the pronotum, the deeper posterior excision of the male sternite VIII and the stouter median lobe of the aedeagus. For illustrations of *D.sextuberculatum* see Shavrin and Smetana (2016).

#### Distribution and natural history.

The type locality is situated in Pailong to the northeast of Linzhi, southern Xizang. The specimens were sifted from leaf litter, moss and mushrooms in broad-leaved forests at an altitude of 2100 m (Fig. 7).

#### Etymology.

Patronymic, named after Xiao-Bin Song, who collected some of the type specimens.

### Deinopteroloma spinigerum

Taxon classificationAnimaliaColeopteraStaphylinidae

Peng & L.-Z. Li sp. n.

http://zoobank.org/AF763E24-11A8-46D2-A04E-DF95595FD2C6

(Figs 4 , 8)

#### Type material.

Holotype ♂: China: Hunan Prov., Yizhang County Mangshan N. R., 24°56'26"N, 112°59'18"E, 26.IV.2017 1400 m, Peng, Tu & Zhou leg. / Holotypus ♂ *Deinopterolomaspinigerum* sp. n., det. Peng & Li, 2019 (SNUC).

#### Description.

Measurements (in mm) and ratios: BL 4.39, FL 3.89, HL 0.57, HW 0.91, AnL 1.61, PL 0.89, PW 1.33, EL 2.34, EW 1.81, AL 1.13, HL/HW 0.63, HW/PW 0.68, HL/PL 0.64, PL/PW 0.67, EL/EW 1.29.

**Figure 4.**
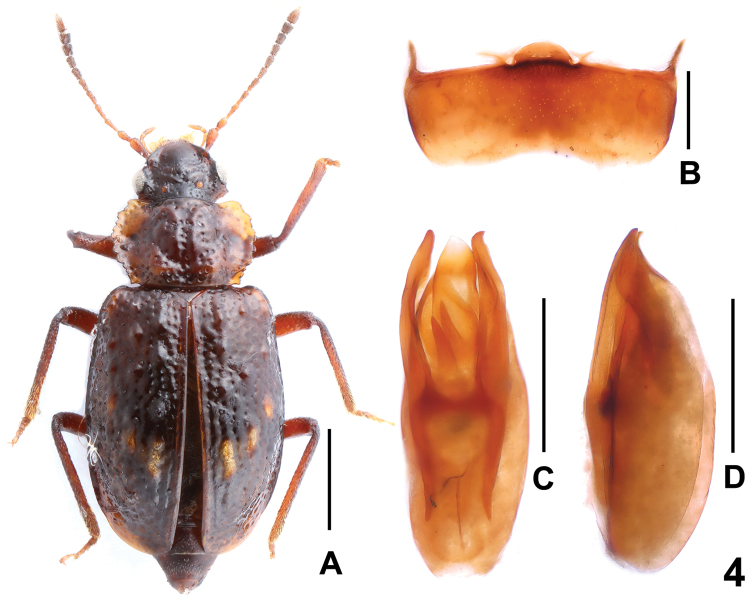
*Deinopterolomaspinigerum* sp. n. **A** Habitus of holotype, **B** male sternite VIII, **C** aedeagus in ventral view, **D** aedeagus in lateral view. Scale bars: 1.0 mm (**A**), 0.5 mm (**B–D**).

Habitus as in Fig. 4A. Coloration: Body dark-brown; pronotum with broadly dark-yellowish lateral margins; each elytron with three yellowish-brown tubercles; antenna with antennomeres I–IV light-brown and V–VI gradually more infuscate apically, VII–XI blackish brown; maxillary palpi dark-yellowish.

Head transverse; posterior part of clypeus and vertex moderately elevated, with indistinct lateral impressions in middle part and weak impression in front of ocelli, infraorbital ridges impressed; eyes large and convex; small acute postocular ridge situated away from posterior margin of eye; ocelli large; frons smooth and glossy; antennomeres I–VIII and XI longer than broad; antennomeres IX–X as long as broad; measurements of antennomeres (length): I: 0.17; II: 0.10; III: 0.12; IV–V: 0.11; VI: 0.07; VII: 0.10; VIII–X: 0.08; XI: 0.14.

Pronotum distinctly transverse; lateral margins finely crenulate and with somewhat irregular outline; lateral portions indistinctly impressed; disc of pronotum with wide, very convex middle elevation and with distinct longitudinal impression; punctation coarse, dense in posteromedian portions, rather sparser in anteromedian and lateral portions, and irregular and scattered in median portion.

Elytra without distinct longitudinal ridges; punctation coarse and arranged in partly irregular series (except in posterior portion of elytra); each elytron with approximately three smooth tubercles. Hind wings probably present.

Male. Abdominal sternites III–VII unmodified; sternite VIII (Fig. 4B) distinctly transverse, posterior margin weakly concave in the middle; aedeagus as in Fig. 4C, D, both parameres with small hook-shaped apex, weakly asymmetric and slender, slightly extending apex of median lobe; internal sac with three sclerotized spines.

#### Female.

Unknown.

#### Comparative notes.

As can be inferred from the similar morphology of the aedeagus, the new species is allied to *D.tricuspidatum* Smetana, 1996 (Fujian: Guadun), from which it differs by the finer punctation of the body, by the presence of three yellowish-brown tubercles on each elytron and the shape of sclerotized spines of the internal sac.

#### Distribution and natural history.

The type locality is situated in Mangshan to the south of Yizhang, southern Hunan. The specimens were sifted from leaf litter in mixed deciduous forests at an altitude of 1400 m (Fig. 8).

**Figures 5–8.**
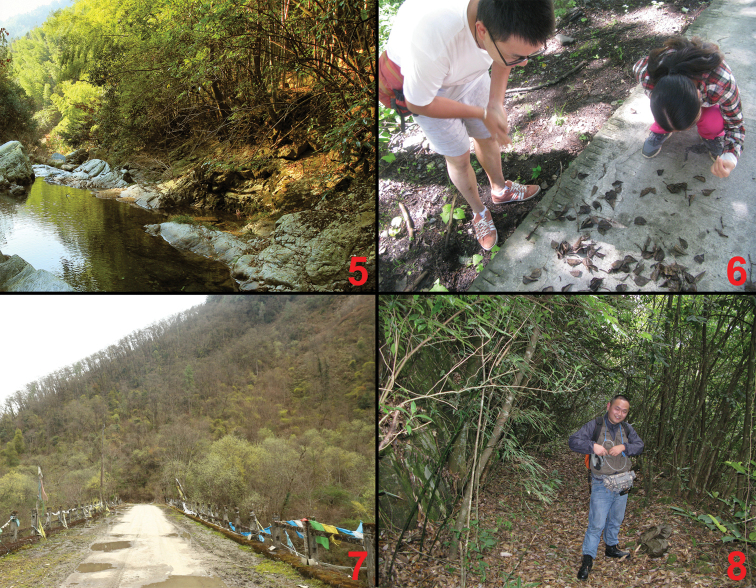
Habitats of *Deinopteroloma* in China: **5** Huang Shan, alt. 460–910 m (*Deinopterolomahamatum*); **6** Sheng-Nan Liu (right) and De-Yao Zhou (left) collecting *Deinopterolomaobtortum* at Jiajin Shan, Sichuan; **7** Pailong, alt. 2100 m (type locality of *Deinopterolomasongi* sp. n.); **8** Ye-Yue Tu collecting *Deinopterolomaspinigerum* sp. n. at Mangshan, Hunan.

#### Etymology.

The specific epithet (Latin, adjective: with spines) alludes to the presence of sclerotized spines in the internal sac of the aedeagus.

### Deinopteroloma tricuspidatum

Taxon classificationAnimaliaColeopteraStaphylinidae

Smetana, 1996

(Fig. 2F, G)

Deinopteroloma tricuspidatum Smetana, 1996: 77.
Deinopteroloma tricuspidatum : Smetana 2001: 57, Shavrin and Smetana, 2016: 227.

#### Type material examined.

Paratype ♀: “KUATUN FUKIEN, China 21.4.46, (TSCHUNG SEN.) / PARATYPUS, *Deinopterolomatricuspidatum*, A. Smetana 1995 [yellow label]” (CNC).

#### Additional material examined.

**China: Zhejiang**: 1 ♀, Qingyuan County, Baishanzu, 1200–1360 m 5.V.2004, Hu, Tang & Zhu leg. (SNUC).

#### Comparative notes.

*Deinopterolomatricuspidatum* was previously known from Guadun, Fujian (Smetana 1996). Considerable interspecific variability of *D.tricuspidatum* was observed in body size, punctation and the shape of pronotum (Fig. 2F, G). The shape of head and the morphology of female genital segment are constant. It is recorded from Zhejiang represents for the first time. For illustrations of *D.tricuspidatum* see Fig. 2F, G and Figs 1–3 in Smetana (1996).

## Supplementary Material

XML Treatment for Deinopteroloma hamatum

XML Treatment for Deinopteroloma obtortum

XML Treatment for Deinopteroloma songi

XML Treatment for Deinopteroloma spinigerum

XML Treatment for Deinopteroloma tricuspidatum
